# Supplementary material for: Institutional decarbonization scenarios evaluated against the Paris Agreement 1.5 °C goal
Source: Nat Commun. 2022 Aug 16;13:4304. doi: 10.1038/s41467-022-31734-1 (PMC9381752; doi:10.1038/s41467-022-31734-1)
Supplement: Supplementary file 1 — Supplementary Information File [file 41467_2022_31734_MOESM1_ESM.pdf]

Supplementary Information: Institutional Decarbonisation Scenarios Evaluated Against the Paris Agreement 1.5°C Goal

Robert J. Brecha[1,2,3*], Gaurav Ganti[1,4*], Robin D. Lamboll[5], Zebedee Nicholls[6,7,8,9], Bill Hare[1], Jared Lewis[7,8,9], Malte Meinshausen[6,7,8], Michiel Schaeffer[1,10], Christopher J. Smith[9,11], Matthew J. Gidden[1,9]

[1] Climate Analytics, Berlin, Germany
[2] Hanley Sustainability Institute, University of Dayton, Dayton, OH, USA
[3] Renewable and Clean Energy Program and Physics Dept., University of Dayton, Dayton, OH, USA
[4] Geography Faculty, Humboldt University, Berlin, Germany
[5] Center for Environmental Policy, Imperial College London, UK
[6] Australian-German Climate and Energy College, The University of Melbourne, Australia
[7] School of Geography, Earth an Atmospheric Sciences, The University of Melbourne, Australia
[8] Climate Resource, Melbourne, Australia
[9] International Institute for Applied Systems Analysis, Laxenburg, Austria
[10] The Global Center on Adaptation, Rotterdam, The Netherlands
[11] Priestley International Centre for Climate, University of Leeds, UK
*Corresponding authors: robert.brecha@climateanalytics.org , gaurav.ganti@climateanalytics.org

Table of Contents

Supplementary Information: Institutional "Paris Agreement Compatible" Mitigation Scenarios Evaluated Against the Paris Agreement 1.5°C Goal 1
1. Evaluating the Constant Quantile Extension (CQE) method 2
2. Evaluating uncertainty due to the infilling method 3
3. Climate assessment – key characteristics of the pathways 5
4. Mitigation lever results 6
5. Applying the FaIR model to assess the climate outcome 7
References 8

1. Evaluating the Constant Quantile Extension (CQE) method

As described in the Methods section of the main text we truncate each pathway (i.e., model and scenario combination) at 2050 and then use the CQE method to extend the pathways to 2100 and calculate the error as:

$$\epsilon = \Sigma_i \sqrt{\Sigma_t \frac{(p_{i,t} - q_{i,t})^2}{n_t\, \sigma_t^2}} / n_i \qquad (1)$$

where ϵ is the error, $p_{i,t}$ is the CQE-extended value of pathway i at time t, $q_{i,t}$ is the originally projected value at that time, $n_{i\ (t)}$ is the number of pathways (times) being summed over and σ_t is the standard deviation of original projections at that time. Supplementary Table 1 shows the relative errors of using the CQE method on the SR1.5 database. It indicates that the errors are generally low - on average 0.23 (0.30 excluding the HFCs), compared to values above 0.5 when using any of the infilling techniques that infer one species from another[1].

Supplementary Table 1: a measure of the average root mean squared errors arising from reconstructing the data in the SR1.5 database after 2050 using the Constant Quantile Extension method, normalised by the standard deviation of the data at each future time, for each emission category.

**Emission Species**	**Relative Error (ϵ)**
BC	0.35805
CH4	0.177191
CO2	0.21437
CO2|AFOLU	0.499959
CO2|Energy and Industrial Processes	0.224759
CO	0.518171
HFC|HFC134a	0.099771
HFC|HFC143a	0.083332
HFC|HFC227ea	0.009422
HFC|HFC23	0.113181
HFC|HFC32	0.158816
HFC|HFC43-10	0.096444
HFC|HFC125	0.083339
N2O	0.265455
NH3	0.148526
NOx	0.325515
OC	0.476686
SF6	0.118234
Sulfur	0.315327
VOC	0.372913

2. Evaluating uncertainty due to the infilling method

Supplementary Table 2: Comparison of multi-gas emission pathways across different infilling methods

Variable	Infill Type	IEA NZE		IEA SDS 2020		BP Net Zero		Shell Sky 1.5		Equinor Rebalance	
		2030	2050	2030	2050	2030	2050	2030	2050	2030	2050
**Energy and Industrial Process CO_2 (Mt CO_2 / yr)**	**Original**	**21283**	**0**	**26824**	**10219**	**28516**	**3685**	**36239**	**19867**	**28853**	**12454**
**AFOLU CO_2 (Mt CO_2 / yr)**	**EQW**	**-1196**	**-7698**	**59**	**-2022**	**309**	**-3897**	**891**	**-5214**	**310**	**-1469**
	**RMS**	**2261**	**1857**	**381**	**-2024**	**-103**	**-4766**	**891**	**-5214**	**1809**	**-1201**
	**QRW**	**19**	**-1272**	**422**	**-1841**	**537**	**-1498**	**891**	**-5214**	**560**	**-1640**
**CH_4 (Mt CH_4 / yr)**	**EQW**	**217**	**112**	**261**	**190**	**275**	**146**	**426**	**281**	**276**	**206**
	**RMS**	**255**	**190**	**259**	**210**	**283**	**214**	**426**	**281**	**234**	**177**
	**QRW**	**248**	**193**	**273**	**199**	**284**	**195**	**426**	**281**	**286**	**203**
**N_2O (kt N_2O / yr)**	**EQW**	**7329**	**5490**	**9250**	**7948**	**9657**	**6596**	**12573**	**11813**	**9657**	**8596**
	**RMS**	**9042**	**9404**	**9460**	**8830**	**9939**	**9149**	**12573**	**11813**	**6841**	**7707**
	**QRW**	**8974**	**8287**	**9674**	**8214**	**9877**	**8175**	**12573**	**11813**	**9917**	**8376**
**BC (Mt BC / yr)**	**EQW**	**5**	**2**	**6**	**4**	**6**	**3**	**7**	**4**	**6**	**4**
	**RMS**	**5**	**5**	**5**	**3**	**7**	**4**	**5**	**3**	**6**	**4**
	**QRW**	**6**	**4**	**6**	**4**	**6**	**4**	**6**	**4**	**6**	**4**
**OC (Mt OC / yr)**	**EQW**	**23**	**8**	**25**	**19**	**27**	**11**	**29**	**21**	**27**	**19**
	**RMS**	**27**	**23**	**23**	**17**	**27**	**20**	**25**	**18**	**28**	**23**
	**QRW**	**28**	**20**	**28**	**20**	**28**	**20**	**29**	**21**	**28**	**20**

There is strong uncertainty in the infilled gases for the Equinor Rebalance and IEA NZE scenarios, depending on the method selected to derive the relationship between the lead gas (CO2 emissions from energy and industrial processes) and the infilled gases. In this section, we evaluate the uncertainty for the CH_4 and N_2O emissions (as an example).

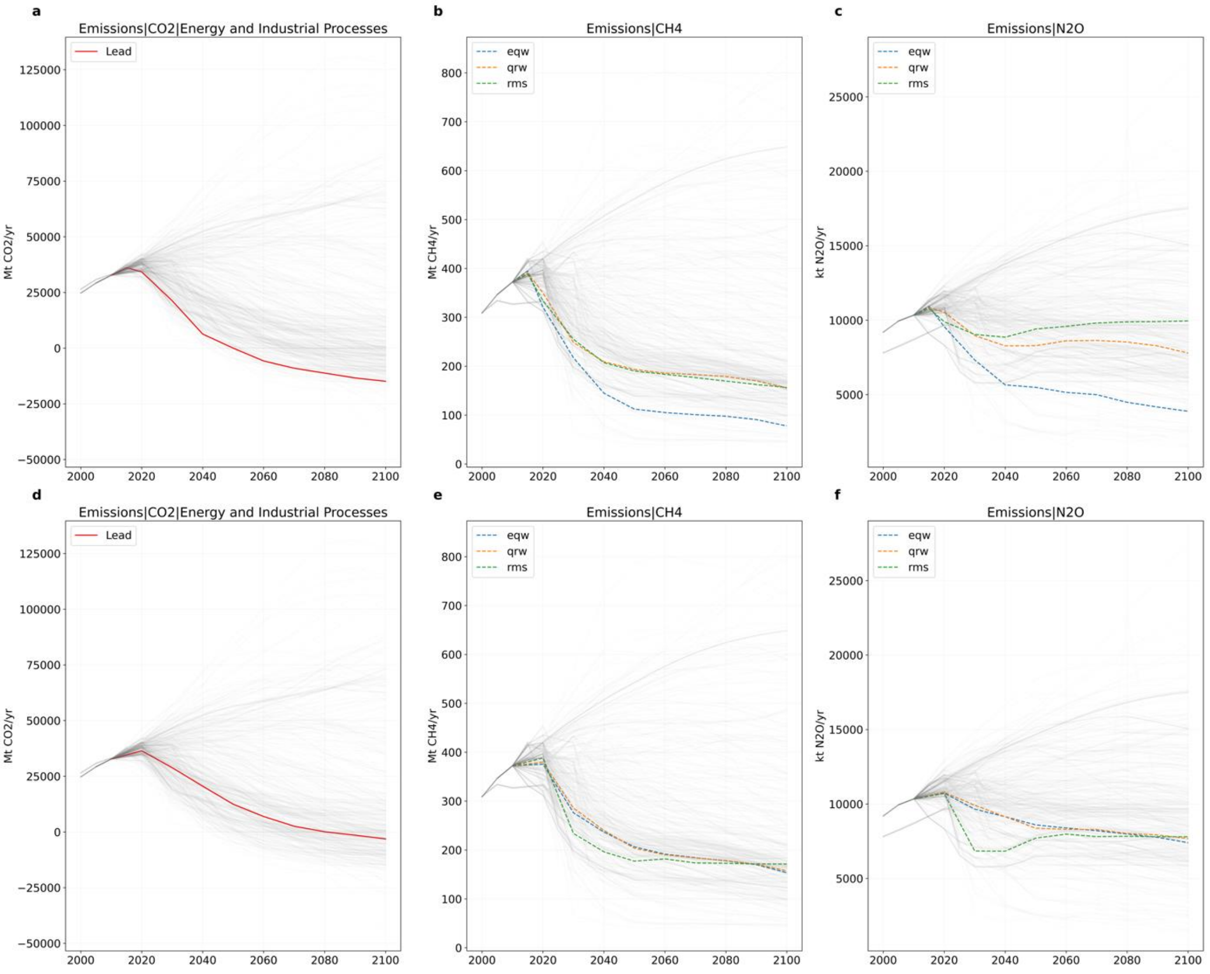

Supplementary Figure 1: Effect of the infilling method for a given lead gas for (a – c) IEA NZE scenario and (d – f) Equinor Rebalance scenario. Thin grey lines represent the scenarios in the SR1.5 database used for infilling.

The RMS pathway selection method can lead to the infilling of relatively extreme emissions that are not necessarily driven by the lead gas. The IEA NZE scenario demonstrates this – while the lead gas emissions drop steeply, the pathway that is selected has relatively high N_2O emissions (*rms* line in Supplementary Figure 1b and c) that indicate a model-specific result. A similar effect is observed for the corresponding emissions for the Equinor Rebalance scenario. On the other hand, the EQW method's inherent assumption of monotonicity can lead to large reductions in the infilled gas without a clear correlation to the lead gas reduction – we see this in the stringent CH_4 reductions in the IEA NZE scenario in panel b (for more details see previously published discussion of the methods[1]). Hence as a default case for the main results, we select the Quantile Rolling Windows (QRW) approach to provide a balanced and consistent approach to infer the missing emissions.

3. Climate assessment – key characteristics of the pathways

The categorisation of pathways on the basis of their climate impact follows the categorisation scheme adopted in SR1.5[2], where categories were constructed based on the probability of exceeding a given temperature target. The categories and their respective exceedance probabilities (P_e) are adapted from SR1.5 and presented in Supplementary Table 3.

Supplementary Table 3: SR1.5 climate categories for pathways

Pathway category	Criteria for assignment
Below 1.5°C	P_e1.5°C <= 0.50
1.5°C low overshoot	0.50 < P_e1.5°C <= 0.67 and P_e1.5°C (2100) <= 0.50
1.5°C high overshoot	0.67 < P_e1.5°C and P_e1.5°C (2100) <= 0.50
Lower 2°C	P_e2.0°C <= 0.34 (excluding above)
Higher 2°C	0.34 < P_e2.0°C <= 0.50
Above 2°C	P_e2.0°C > 0.50

The first climate outcome that is necessary (but not sufficient) to assess the Paris compatibility of the different pathways, is to check whether they achieve a balance between sources and sinks of emissions in the second half of the century. In Supplementary Table 4, we report the total greenhouse gas pathway (infilled using the QRW method) for the institutional scenarios.

Supplementary Table 4: Total Kyoto greenhouse gas emissions per scenario (infilled using the QRW method)

Scenario	Unit	2020	2030	2040	2050	2060	2070	2080	2090	2100
**IEA (NZE)**	Mt CO2-equiv/yr	49578	30254	13349	6072	-748	-3828	-5765	-8376	-10750
**BP (Net Zero)**	Mt CO2-equiv/yr	53444	39157	18854	9520	3342	649.4	-2894	-4593	-7047
**BP (Rapid)**	Mt CO2-equiv/yr	53444	42085	26648	17366	10372	6639	4830	2618	613.8
**Equinor (Re-balance)**	Mt CO2-equiv/yr	52796	39581	28737	18412	11824	7560	5158	3349	1103
**IEA (SDS)**	Mt CO2-equiv/yr	53158	37022	24488	15818	9119	5371	3276	1149	-1356
**Shell (Sky)**	Mt CO2-equiv/yr	51833	51718	42817	25274	6049	-910	-3876	-3671	-3325

In Supplementary Table 5 we present the variation of the climate categorization across the different infilling methods assessed in this study. In only one case is there a change in categorization due to infilling method because the scenario results lie near the boundary between two categories.

Supplementary Table 5: Categorisation across different infilling methods

Scenario/IF method	QRW	RMS	EQW
**IEA (NZE)**	1.5C low overshoot	1.5C high overshoot	1.5C low overshoot
**BP (Net Zero)**	1.5C high overshoot	1.5C high overshoot	1.5C high overshoot
**BP (Rapid)**	Lower 2C	Lower 2C	Lower 2C
**Equinor (Rebalance)**	Lower 2C	Lower 2C	Lower 2C
**IEA (SDS)**	Lower 2C	Lower 2C	Lower 2C
**Shell (Sky)**	Lower 2C	Lower 2C	Lower 2C

4. Mitigation lever results

In Supplementary Table 6 we summarise results for two technology mitigation levers, as discussed in the main text. The total final energy demand (E_t) and the carbon intensity of final energy (CI_t) are given as relative levels compared to 2010 for pathways of various climate outcome categories as derived from the SR1.5, as well as for the scenarios analysed in the present work.

Supplementary Table 6: Comparison of mitigation levers

**Mitigation Lever**	**Pathway**	**Source**	**Level compared to 2010 (%) Median (Interquartile Range)**	
			**2030**	**2050**
**E_t**	**Below 1.5C [7]**	**SR15**	**84 (96, 82)**	**89 (112, 85)**
	**1.5C low overshoot [43]**	**SR15**	**103 (109, 92)**	**112 (122, 95)**
	**1.5C high overshoot [35]**	**SR15**	**125 (129, 118)**	**138 (147, 120)**
	**Lower 2C [74]**	**SR15**	**113 (126, 103)**	**127 (138, 112)**
	**Higher 2C [58]**	**SR15**	**124 (133, 112)**	**144 (151, 122)**
	**Above 2C [188]**	**SR15**	**134 (143, 127)**	**160 (178, 148)**
	**Rebalance**	**Equinor**	**113**	**98**
	**Sky**	**Shell**	**124**	**149**
	**SDS**	**IEA**	**109**	
	**NZE2050**	**IEA**	**106**	**93**
**CI_t**	**Below 1.5C [7]**	**SR15**	**47 (50, 42)**	**4 (10, 3)**
	**1.5C low overshoot [43]**	**SR15**	**62 (72, 57)**	**11 (18, -0)**
	**1.5C high overshoot [35]**	**SR15**	**73 (82, 66)**	**9 (22, 3)**
	**Lower 2C [74]**	**SR15**	**71 (81, 61)**	**29 (34, 23)**
	**Higher 2C [58]**	**SR15**	**80 (82, 74)**	**38 (45, 28)**
	**Above 2C [188]**	**SR15**	**94 (103, 88)**	**84 (98, 61)**
	**Rebalance**	**Equinor**	**79**	**40**
	**Sky 1.5**	**Shell**	**89**	**40**
	**SDS**	**IEA**	**76**	
	**NZE**	**IEA**	**62**	**0**

5. Applying the FaIR model to assess the climate outcome

In this section, we demonstrate the application of the FaIR model[3,4] to assess the climate outcome of the institutional scenarios. For this sensitivity, we perform the climate assessment across all infilling sensitivity cases, and then calculate the average difference between the MAGICC6 and FaIR results. Importantly, we use the same priors (parameter sets) that were used in SR1.5 while performing the FaIR assessment. SR1.5 noted the following, with respect to the comparison between MAGICC6 and FaIR results: "The comparison of these lines of evidence shows *high agreement* in the relative temperature response of pathways, with *medium agreement* on the precise absolute magnitude of warming, introducing a level of imprecision in these attributes."

Since the purpose of this paper is to assess the climate outcome of institutional pathways, in line with the assessment in SR1.5 (which kept consistency with AR5), we would expect the same outcome to be reflected in our results for the institutional pathways. In Supplementary Table 7, we highlight the difference in peak and end-of-century warming (in both cases we compare the median of the probabilistic temperature distributions) between the MAGICC6, and FaIR setups. A positive value indicates that MAGICC6 has higher values than the corresponding FaIR values. We assess the difference for 18 scenarios (i.e., 6 institutional scenarios, infilled with 3 different methods).

Supplementary Table 7: Difference between FaIR and MAGICC results

	n	Mean	Standard Deviation
Difference in peak warming (°C)	18	0.25	0.02
Difference in end of century warming (°C)	18	0.18	0.03

Supplementary Note

The institutions associated with the scenarios considered in this manuscript were contacted for a response to the manuscript. Equinor had no comment, and we received no response from BP or Shell. IEA requested clarifications, which were addressed in the manuscript.

Supplementary References

1. Lamboll, R. D., Nicholls, Z. R. J., Kikstra, J. S., Meinshausen, M. & Rogelj, J. Silicone v1.0.0: An open-source Python package for inferring missing emissions data for climate change research. *Geosci. Model Dev.* **13**, 5259–5275 (2020).
2. Rogelj, J. *et al.* Chapter 2: Mitigation Pathways Compatible with 1.5°C in the Context of Sustainable Development. in *Global Warming of 1.5°C. An IPCC Special Report on the impacts of global warming of 1.5°C above pre-industrial levels and related global greenhouse gas emission pathways, in the context of strengthening the global response to the threat of climate change* (eds. Masson-Delmotte, V. et al.) (IPCC, 2018).
3. Smith, C. J. *et al.* FAIR v1.3: A simple emissions-based impulse response and carbon cycle model. *Geosci. Model Dev.* **11**, 2273–2297 (2018).
4. Millar, J. R., Nicholls, Z. R., Friedlingstein, P. & Allen, M. R. A modified impulse-response representation of the global near-surface air temperature and atmospheric concentration response to carbon dioxide emissions. *Atmos. Chem. Phys.* **17**, 7213–7228 (2017).
