# Supplementary material for: Institutional decarbonization scenarios evaluated against the Paris Agreement 1.5 °C goal
Source: Nat Commun. 2022 Aug 16;13:4304. doi: 10.1038/s41467-022-31734-1 (PMC9381752; doi:10.1038/s41467-022-31734-1)
Supplement: Supplementary file 3 — Reporting Summary [file 41467_2022_31734_MOESM3_ESM.pdf]

nature portfolio

Corresponding author(s): Robert J. Brecha, Gaurav Ganti

Last updated by author(s): 2021-11-08

# Reporting Summary

Nature Portfolio wishes to improve the reproducibility of the work that we publish. This form provides structure for consistency and transparency in reporting. For further information on Nature Portfolio policies, see our Editorial Policies and the Editorial Policy Checklist.

## Statistics

For all statistical analyses, confirm that the following items are present in the figure legend, table legend, main text, or Methods section.

| n/a | Confirmed | |
|---|---|---|
| ☐ | ☒ | The exact sample size (*n*) for each experimental group/condition, given as a discrete number and unit of measurement |
| ☒ | ☐ | A statement on whether measurements were taken from distinct samples or whether the same sample was measured repeatedly |
| ☒ | ☐ | The statistical test(s) used AND whether they are one- or two-sided<br>*Only common tests should be described solely by name; describe more complex techniques in the Methods section.* |
| ☒ | ☐ | A description of all covariates tested |
| ☒ | ☐ | A description of any assumptions or corrections, such as tests of normality and adjustment for multiple comparisons |
| ☒ | ☐ | A full description of the statistical parameters including central tendency (e.g. means) or other basic estimates (e.g. regression coefficient) AND variation (e.g. standard deviation) or associated estimates of uncertainty (e.g. confidence intervals) |
| ☒ | ☐ | For null hypothesis testing, the test statistic (e.g. *F*, *t*, *r*) with confidence intervals, effect sizes, degrees of freedom and *P* value noted<br>*Give P values as exact values whenever suitable.* |
| ☒ | ☐ | For Bayesian analysis, information on the choice of priors and Markov chain Monte Carlo settings |
| ☒ | ☐ | For hierarchical and complex designs, identification of the appropriate level for tests and full reporting of outcomes |
| ☒ | ☐ | Estimates of effect sizes (e.g. Cohen's *d*, Pearson's *r*), indicating how they were calculated |

*Our web collection on statistics for biologists contains articles on many of the points above.*

## Software and code

Policy information about availability of computer code

| | |
|---|---|
| Data collection | No software was used for collection of the institutional scenario data<br>The scenario data from the Special Report on 1.5C were collected using the interface provided by the open source Python package pyam (details in the next field) |
| Data analysis | The software and scripts used in this study are available in a gitlab repository at https://gitlab.com/gaurav-ganti/institutional_scenarios<br>The environment .yml file in this repository documents the python packages and versions that are necessary for this analysis. |

For manuscripts utilizing custom algorithms or software that are central to the research but not yet described in published literature, software must be made available to editors and reviewers. We strongly encourage code deposition in a community repository (e.g. GitHub). See the Nature Portfolio guidelines for submitting code & software for further information.

## Data

Policy information about availability of data

All manuscripts must include a data availability statement. This statement should provide the following information, where applicable:

- Accession codes, unique identifiers, or web links for publicly available datasets
- A description of any restrictions on data availability
- For clinical datasets or third party data, please ensure that the statement adheres to our policy

The input scenarios data that support the findings of this study are available on reasonable request from the corresponding author due to license restrictions from the data providers.

# Field-specific reporting

Please select the one below that is the best fit for your research. If you are not sure, read the appropriate sections before making your selection.

☐ Life sciences ☐ Behavioural & social sciences ☒ Ecological, evolutionary & environmental sciences

For a reference copy of the document with all sections, see nature.com/documents/nr-reporting-summary-flat.pdf

# Ecological, evolutionary & environmental sciences study design

All studies must disclose on these points even when the disclosure is negative.

| | |
|---|---|
| Study description | A uniform and transparent methodology is employed to evaluate Paris Agreement compatibility of influential "institutional" emission scenarios from the grey literature, including those from Shell, BP, and the IEA. The methodology enables like-for-like comparisons of climate variable implications for any emissions pathways, given sufficient emissions data. |
| Research sample | The main research sample are emissions scenarios published by major institutions. The comparison sample are those used for IPCC assessments and from the integrated assessment literature. |
| Sampling strategy | All available institutional pathways are evaluated. |
| Data collection | The input institutional scenario data is available on reasonable request due to license restrictions.<br>The Special Report on 1.5°C is openly available at https://data.ene.iiasa.ac.at/iamc-1.5c-explorer/#/login?redirect=%2Fworkspaces |
| Timing and spatial scale | Temporal scale of the investigation is fundamentally to evaluate pathways and climate variables to the end of the century.<br>Methodologies are explained for extending those data sets that only provide emissions for a shorter period |
| Data exclusions | Data were not excluded |
| Reproducibility | The "pipeline" of assessment of scenarios is available so that results may be reproduced by other users |
| Randomization | Randomization was not necessary as we selected all available data sets from institutions who have published trajectories claimed to be consistent with the Paris Agreement |
| Blinding | No blinding was used or necessary for this study of model output data |

Did the study involve field work? ☐ Yes ☒ No

# Reporting for specific materials, systems and methods

We require information from authors about some types of materials, experimental systems and methods used in many studies. Here, indicate whether each material, system or method listed is relevant to your study. If you are not sure if a list item applies to your research, read the appropriate section before selecting a response.

## Materials & experimental systems

| n/a | Involved in the study |
|---|---|
| ☒ | ☐ Antibodies |
| ☒ | ☐ Eukaryotic cell lines |
| ☒ | ☐ Palaeontology and archaeology |
| ☒ | ☐ Animals and other organisms |
| ☒ | ☐ Human research participants |
| ☒ | ☐ Clinical data |
| ☒ | ☐ Dual use research of concern |

## Methods

| n/a | Involved in the study |
|---|---|
| ☒ | ☐ ChIP-seq |
| ☒ | ☐ Flow cytometry |
| ☒ | ☐ MRI-based neuroimaging |
